# Exploring Neural Signaling Patterns and Their Physiological Origins in Fibromyalgia by Means of Functional MRI Guided by a Review of the Literature

**DOI:** 10.3390/brainsci15060603

**Published:** 2025-06-04

**Authors:** Mara Will, Patrick W. Stroman

**Affiliations:** Centre for Neuroscience Studies, Queen’s University, Kingston, ON K7L 3N6, Canada; 20mgw@queensu.ca

**Keywords:** fibromyalgia, functional MRI, analysis, review, pain, brain, brainstem, physiology

## Abstract

Background/Objectives: Fibromyalgia (FM) is a chronic pain condition that includes symptoms of hyperalgesia and has an unknown etiology. This study aimed to further investigate the underlying neural signaling mechanisms and their relation to observed blood oxygenation-level dependent (BOLD) signal increases at the onset of functional magnetic resonance imaging (fMRI) runs. Methods: The possible neural mechanisms were first explored by reviewing the current literature. The second component of this study involved a voxel-by-voxel analysis of BOLD responses in all regions of the brain. The fMRI data were obtained from a previous study of participants with and without fibromyalgia during fMRI runs involving either a noxious heat pain stimulus or no stimulus. Results: The literature review indicates that no single factor can explain the initial BOLD signal rise observed in FM but that there are likely multiple interacting influences. These include physiological dysregulation via mechanisms, such as oxidative stress, mitochondrial dysfunction, and cytokine activity, and may involve the sympathetic nervous system. The analysis of BOLD responses demonstrated that the initial BOLD rises occur specifically in gray matter regions and are largest in regions involved with pain processing, including the right insular cortex and periaqueductal gray region. Moreover, the BOLD rise is significantly larger in people with FM prior to the application of a noxious stimulus. Conclusions: The initial rise in BOLD response demonstrates heightened metabolic demand that is exaggerated in people with FM. It appears to be influenced by cognitive factors such as anticipation and may reflect neural dysregulation, possibly involving autonomic signaling.

## 1. Introduction

Fibromyalgia (FM) is a prevalent chronic pain disorder with, as of yet, unknown etiology. Extensive research has explored both central and peripheral neural mechanisms that may contribute to FM symptomatology. Despite these efforts, no single explanation has been identified regarding the disorder’s onset and progression or the heightened nociceptive responses that occur. Functional magnetic resonance imaging (fMRI) studies of FM have consistently demonstrated altered neural signaling in response to noxious stimuli but have not revealed the underlying mechanisms [1,2,3,4,5,6,7,8,9,10,11,12,13,14,15,16,17,18,19,20,21]. For example, regions such as the anterior cingulate cortex (ACC), posterior cingulate cortex (PCC), insular cortex (IC), and thalamus have frequently been identified in fMRI studies as exhibiting irregular blood oxygenation-level dependent (BOLD) signals or altered connectivity between regions [22]. Recently, a novel feature of FM has been reported in the form of a significant rise in BOLD signal at the onset of fMRI runs prior to the application of a stimulus [23]. This initial rise was observed but not significant in healthy control (HC) participants, and its magnitude is much larger in FM. The BOLD signal increase was observed to occur within the first 30 s of every fMRI run, indicating heightened metabolic demand within cortical regions, that is, sustained throughout the remainder of each fMRI run. However, the metabolic demand appears to return to a lower baseline state between runs, resulting in an observed increase again at the onset of each subsequent run. The BOLD increase thus appears to reflect a heightened state of activity across multiple brain regions. These findings prompt the questions of the cause of the initial rise in BOLD signal in FM, how or if it relates to altered pain processing in FM, and whether or not related effects have been reported previously in the literature.

Previous research that has not involved neuroimaging has explored various pathophysiological mechanisms that may contribute to our understanding of FM and the findings from fMRI. It is understood that pain is perceived as a result of ascending and descending neural pathways, in which nociceptive signals in peripheral afferent neurons travel through the spinal cord to the brainstem and to brain regions. Cortical regions process and interpret the signals and result in the experience of pain [24]. However, descending pathways from the brainstem can modify these pain signals before they reach the brain [24]. This system relies on neurotransmitters, including serotonin, dopamine, and noradrenaline, which can influence the intensity of pain [24]. As a result, chronic pain disorders such as FM may occur as a result of irregularities in these neural pathways or neurotransmitter processing. Several potential mechanisms of altered neural signaling in FM have been proposed, including increased brain-derived neurotrophic factor (BDNF) serum levels [25], deoxyribonucleic acid (DNA) methylation mutations [26], dopamine (DA) dysregulation [27], mitochondrial dysfunction [28], increased cytokine presence [29], and irregular glial cell activity [25]. However, no singular explanation consistent throughout this research provides evidence of how FM arises and what psychological or physiological mechanisms may be responsible for its symptoms.

In the present study, we aimed to advance our understanding of FM by investigating the initial rise in BOLD response by means of a focused review of the literature and new analyses of existing fMRI data [23]. Previous research on the etiology of fibromyalgia (FM) has investigated singular pathophysiological mechanisms that contribute to FM symptomology and onset, or abnormalities in neural signaling and brain structure, as observed through imaging techniques like fMRI. However, these studies rarely integrated both aspects to explain not only what is occurring in FM but also why these processes occur. The literature review in the first part of the present study was therefore aimed specifically at identifying existing evidence of physiological or psychological mechanisms that could explain the initial rise in BOLD signal. The second part of this study involved quantitative data analyses to further explore the anatomical and temporal characteristics of the initial rise in BOLD signal in FM compared to HC participants. Based on the prior evidence, we hypothesized that (a) the BOLD signal increase occurs in pain-related regions, (b) is localized within grey matter, and (c) is larger in people with FM.

## 2. Materials and Methods

The methods used for this study relate to the two components: (1) a literature search in order to identify possible explanations for the initial rise in BOLD response that have already been identified in other research studies and (2) a detailed analysis of existing BOLD fMRI data from participants with FM, as well as an age- and sex-matched group of healthy control participants.

### 2.1. Component 1: Literature Review

**Figure 1:**
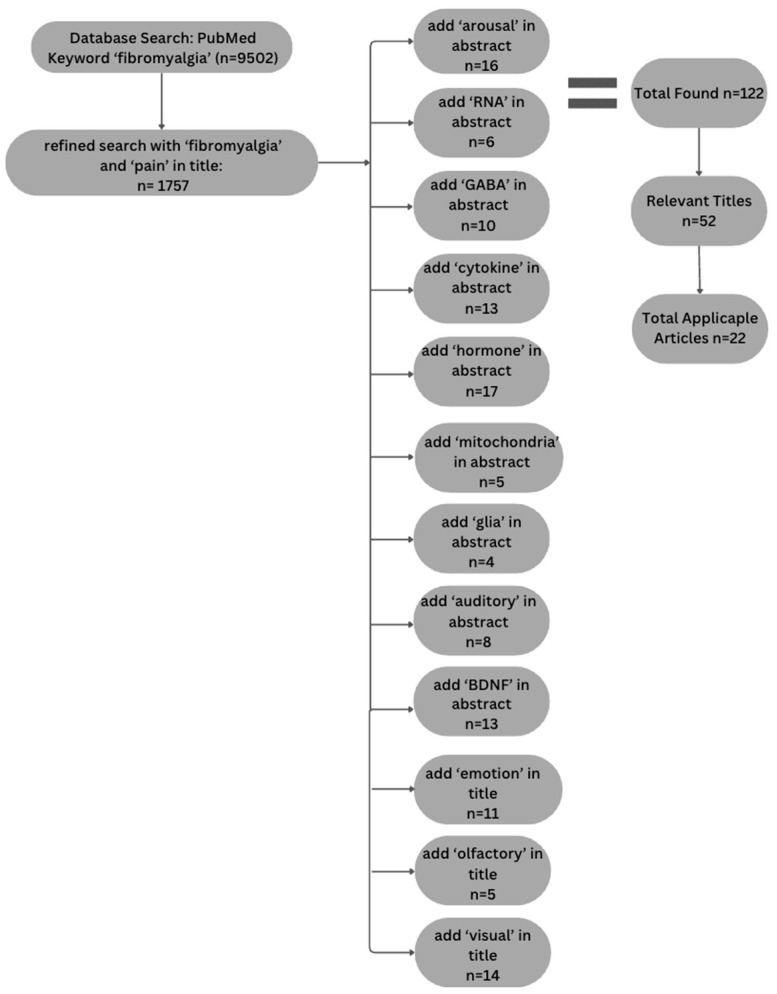
Flow chart depicting article selection process used to conduct literature analysis following PRISMA guidelines.

A systematic literature search was conducted to explore the physiological and psychological mechanisms contributing to increased arousal in FM. The purpose of the review was focused on identifying prior studies that might provide information about the initial rise in BOLD signal, as opposed to being a comprehensive review of all studies of FM. The search was performed using the PubMed database. The selection process adhered to the Preferred Reporting Items for Systematic Reviews and Meta-Analyses (PRISMA) guidelines and is depicted in Figure 1.

Initially, broad search terms such as “fibromyalgia” and “pain” were used in the title, yielding 1757 articles. To refine the results, articles were selected that included one or more of the following terms in the abstract field: “BDNF”, “GABA”, “arousal”, “auditory”, “mitochondria”, “glia”, “cytokines”, “RNA”, or “hormone”. Articles were also included that had the terms “emotion”, “olfactory” and “visual” in the title because including these terms in the abstract did not adequately narrow the search. With these limits to the search terms, the number of articles was reduced to 122. We did not include terms such as “fMRI” or “MRI” because the focus of this review was to identify possible physiological or psychological mechanisms that could explain the observed initial rise in BOLD response.

Study eligibility was determined by means of an initial title screening, followed by an abstract review of these studies, further reducing the selection to 52 articles. Studies were included and deemed relevant if they investigated FM while focusing on pain and pain perception, particularly exploring etiology and heightened arousal. Additionally, inclusion criteria consisted of articles that focused on the selected search terms and did not explore additional topics. Studies were excluded if FM was not the primary focus, if they explored other forms of chronic pain conditions, or if the primary issue addressed revolved around treatment rather than etiology. If the study did not align with the specific search terms used to refine articles within the database as well, they were excluded from the literature search. No restrictions were placed on study design, meaning randomized controlled trials, observational studies, and review articles were all considered. Articles were limited to those published in English, but no restrictions were placed on the publication date, as most relevant articles were published within the last 20 years. A full-text review further refined the pool to 19 articles thought to be appropriate and reliable for inclusion in the literature review. These 19 articles were ultimately included based on their relevance to pain perception, heightened arousal, and alignment with the selected search topics.

Studies were then categorized for synthesis by grouping the relevant articles according to whether they discuss physiological or psychological mechanisms. These were further subcategorized based on their primary focus such as emotions, auditory processing, genetic factors and methylation, neurotransmitters, proteins, mitochondria, glia, and hormones.

### 2.2. Component 2: Analysis of Existing fMRI Data

#### 2.2.1. Source of the Existing Data and Supporting Participant Information

Data for this study were obtained from a previous study carried out in the Stroman lab, as reported by Warren et al. [22]. This study provided the background research and rationale for the present study and provided complete brain fMRI data sets from healthy women as well as women diagnosed with FM (20 FM and 17 HC). In addition to the fMRI data, questionnaire data and ratings of the pain intensity and unpleasantness experienced during each fMRI run were available in order to identify relationships between pain behaviors, psychological characteristics, and fMRI measures.

Questionnaire data provide information about psychological factors such as anxiety, depression, social influence, and pain catastrophizing and also information about contributions of altered autonomic function on pain processing [22]. The questionnaires used to measure these traits included the State–Trait Anxiety Inventory (STAI), Beck Depression Inventory-II (BDI-II), Composite Autonomic Symptom Score 31 (COMPASS-31), Social Desirability Scale (SDS), Pain Catastrophizing Scale (PCS), the Revised Fibromyalgia Impact Questionnaire (FIQR) and the Short-Form Pain Questionnaire-2 (SF-MPQ-2). Participants were also tested to determine whether they met the current criteria for an FM diagnosis based on the 2016 Fibromyalgia Survey Questionnaire (FSQ).

Participants first underwent a one-hour-long training session in order to become accustomed to the fMRI environment and the study procedures [22]. This included being familiarized with the use of a standardized numerical pain intensity scale (NPS) (0 being no pain and 100 being intolerable) to rate their pain. The device used throughout the study to administer noxious stimuli was a custom-made MRI-compatible robotic contact-heat thermal stimulator (RTS-2). During the study and training sessions, the RTS-2 contacted the thenar eminence of the right hand. To become accustomed to it, participants experienced three short heat contacts at 45, 47, and 46 °C, within durations of 1.5 s and onsets every 3 s. Participants were then given four calibration tests that involved 10 consecutive heat contacts over 30 s at temperatures of 46, 50, 44, and 48 °C (unless the temperature had to be lowered due to intense pain in FM participants). These calibration tests were then used to determine the temperature to be used during the fMRI experiment in order to elicit moderate pain (approximately 50/100) in each participant. Participants additionally underwent a practice fMRI run in a sham MRI scanner at the end of the training session. Participants were instructed to silently rate their pain in response to each contact and to remember their ratings for the first and last contacts.

**Figure 2:**
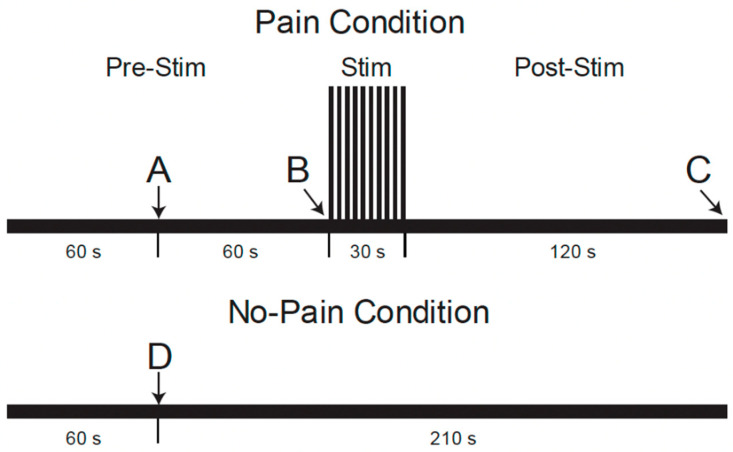
Functional MRI paradigm for conditions with and without noxious heat stimulation conditions [22]. The timeline shows when participants were told whether or not they would experience the painful condition or not at time points A and D, when the noxious stimulus was administered at point B, and when the participants were asked to voice their pain ratings to the first and last heat contacts at point C.

During the MRI data acquisition portion of the study, repeated fMRI runs were applied with two different conditions, “Pain” or “No-Pain”, in a pseudo-randomized order [22]. As shown in Figure 2, (reproduced from Warren et al. [22]), fMRI paradigms were 4.5 min long and involved a period of noxious heat stimulation in the “Pain” condition, but no stimulus was applied in “No-Pain” runs. Participants were informed 1 min after the start of each run whether or not to expect the heat stimulus to be applied. For the “Pain” condition, the repeated heat contacts (1.5 s duration, 3 s between contacts) were applied starting at the 2 min mark for a duration of 30 s [22]. For the “No-Pain” condition, no stimulus was applied. After the stimulation period (or no-stimulation), the data acquisition was continued for a total of 4.5 min for both conditions. After each run, participants were asked to verbally report their pain ratings for the first and last heat contacts, as practiced during the training session.

#### 2.2.2. Analysis Methods for the Present Study

Data analysis was completed by means of custom-written software written in Python 3.8, using the previously collected, pre-processed data. For each of the FM and HC groups, the two conditions are referred to as “stim” and “rest” for the conditions with and without a stimulus applied, respectively. The results are therefore presented for four group/condition combinations: HC stim, HC rest, FM stim, and FM rest. Data were obtained from 17 participants with FM, with a total of 84 fMRI runs in the stim condition and 79 runs in the rest condition. Data were also obtained from 15 HC participants, with 63 fMRI runs in the stim condition and 66 in the rest condition.

The fMRI data were obtained at 3 tesla, using a gradient-echo EPI sequence, with 2 × 2 × 2 mm^3^ voxels, with an echo time (TE) of 30 ms for optimal BOLD sensitivity, and a repetition time (TR) of 2 s, for a total of 135 time points to describe the fMRI time-series responses for each run [22]. As the data sets had been previously analyzed, they were already pre-processed. This included excluding the first 3 time points to avoid non-steady-state effects, motion correction, slice timing correction, and spatial normalization to the MNI152 template after interpolation to 1 mm cubic voxels. All preprocessing was performed using custom-written functions in Python in the software package “Pantheon” (available online: https://github.com/stromanp/pantheon-fMRI (accessed on 29 May 2025)).

**Figure 3:**
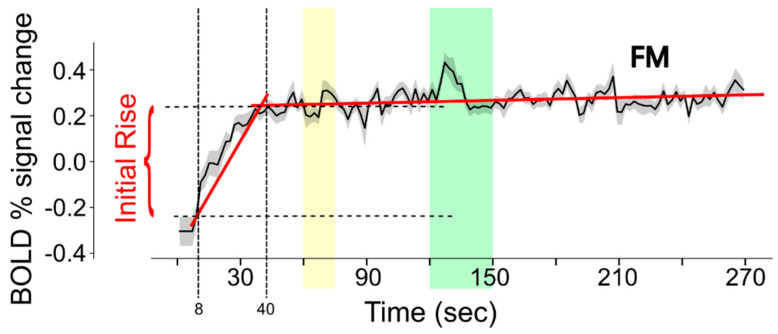
Illustration of the method used to estimate the initial rise in BOLD response in each voxel of each fMRI run. BOLD signal change values are fit to linear functions for the periods 8–40 s and 40–270 s (the fits are shown with red lines) in order to reliably determine the intensity values at 8 s and 40 s (indicated with vertical dashed lines). The horizontal dashed lines indicate the signal values at these times, for the example shown. Note that the data shown are the average across the entire FM study group and that actual data from each participant are much noisier than is depicted in this example. The yellow band indicates the period when participants were informed of the stimulus type to expect, and the green band indicates the stimulation period.

For the current analysis, data were first resized to 4 mm cubic voxels. The purpose of this step was to reduce the number of voxels in the analysis and to increase the signal-to-noise ratio. The BOLD fMRI time-series data in each voxel were analyzed to characterize the signal change during the first 40 s of each run, as depicted in Figure 3. The data within the time span of 8 s to 40 s (time period 1) were fit to a linear function. The data within the time span of 40 s to 270 s (time period 2) were fit to a separate linear function. The intercept value for time period 1 demonstrates the fit signal intensity at the 8 s time point. The signal intensity at the 40 s time point was determined from the linear fit to time period 2. The initial rise in BOLD response was quantified by the difference between the fit values at 40 s and at 8 s.

The results obtained from all fMRI runs were combined across each group by computing the average and the standard deviation of the measures in each voxel. Images depicting the anatomical distribution of the initial rise values above a selected magnitude threshold of 0.25% were then created. The average initial rise values were converted to a red–green–blue color scale spanning the range of measured values. The color values were superimposed on an anatomical reference image represented with a grey scale, only for the voxels with initial rise magnitudes that exceeded the threshold.

As discussed below, the voxel-wise results highlighted several cortical regions that are involved with multiple functions, including pain processing. Average signal rise values were therefore also determined for anatomical regions, which were selected based on their known involvement with processing pain and nociceptive signaling. The selected regions included the insular cortex (IC), posterior cingulate cortex (PC), anterior cingulate cortex (AC), frontal orbital region (fOrb), periaqueductal gray matter region (PAG), the hypothalamus, thalamus, parabrachial nuclei (PBN), and the amygdala. These regions were identified in spatially normalized fMRI data based on the anatomical region map in the Pantheon software package [30,31]. This anatomical map is based largely on the anatomical map included in the CONN15e software package supplemented by other freely shared anatomical maps and descriptions [32,33,34,35,36,37,38,39,40,41,42,43].

## 3. Results

### 3.1. Study Component 1: Literature Review

The results of the literature review are summarized as follows, divided into key topics of Cognitive Influences and Physiological Influences, as well as sub-topics within each of these areas.

#### 3.1.1. Cognitive Influences

Although FM is a chronic pain disorder characterized by physical symptoms, studies have explored the possibility of underlying cognitive differences amongst individuals with this disorder compared to healthy controls. This may be observed through the diagnoses of depression and anxiety disorders influencing their overall neural processing and arousal. Another potential factor is increased sensitivity to external sensory stimuli, affecting how sensory information is processed within these individuals. In this literature review, the idea of both increased emotional arousal and sensitivity to auditory stimuli is explored.

##### Emotional Arousal

Several studies have explored the relationship between FM and its comorbidity with psychological disorders. Since prior research has indicated that FM may involve both central and peripheral mechanisms, it has been questioned whether emotional sensitization could contribute to the nociceptive dysfunction observed in individuals with this condition [44]. Previous studies have explored whether an individual’s ability to regulate emotions may help them adapt to chronic pain, as negative emotions have been correlated with increased pain, sensitivity, cognitive deficits, and additional psychological disorders that diminish quality of life [44]. Based on this information, researchers have explored the possibility that poor cognitive regulation and negative emotions could affect the neurobiological processes underlying FM, potentially explaining why individuals experience chronic pain. For instance, this can be seen as the stress caused by chronic pain increasing the production and release of glutamate, which in turn, may contribute to emotional dysregulation [44]. To test this theory, Ciuffini et al. [44] investigated the emotional arousal and emotional valence responses of FM patients when exposed to unpleasant stimuli. This study concluded that they exhibited heightened activation in response to negative emotional stimuli, demonstrating increased levels of emotional arousal. These results suggested that the mechanisms of central sensitization and neural regulation may be influenced by abnormal arousal, with negative emotions acting as a stressor. Increased sensitization in the form of emotional arousal can be associated with the reactivity of the anterior cingulate cortex and the insula, both of which are associated with the emotional experience of pain that is thought to be regulated within the limbic system. It is also possible that an individual’s cognitive relationship with pain may influence their emotional sensitivity and overall experience with central and peripheral stimuli. Simply put, the way an individual with FM perceives pain, in conjunction with their body’s increasing sensitivity to nociceptive stimuli over time, may explain how this condition manifests differently amongst individuals. However, it can be questioned whether the nociceptive dysfunction experienced in FM is initiated by increased emotional sensitization or if this increased sensitivity is a reflection of the discomfort and suffering these individuals experience daily. This distinction is difficult to quantify, as being in constant pain without an identifiable cause is in itself a possible cause of increased psychological distress and sensitivity.

As previously mentioned, individuals with FM experience different forms of emotional arousal and maladaptive cognitive–emotional responses, which the high comorbidity of psychological disorders such as depression and anxiety may influence. This raises the question of whether the heightened arousal observed in FM participants is in part a result of emotional dysregulation. Frumer et al. [45] found that maladaptive emotional responses are associated with FM symptoms and its comorbidity with stress and depression. It is therefore suggested that difficulties in emotion regulation could explain the existence of psychological conditions in FM [45].

Furthermore, the relationship between pain catastrophizing and depression has been explored as a contributing factor to heightened arousal and maladaptive cognitive-emotional responses within FM [46]. Through assessing the varying psychological elements of depression, fatigue, and pain catastrophizing, these variables were found to predict reduced functional capacity within FM, which were determined to be associated with increased pain intensity and functional impairment [46]. These findings highlight the significant relationship between the maladaptive emotional responses present in FM symptomology and their relationship to the nociceptive symptoms. Taken together, these cognitive-emotional factors, alongside physiological mechanisms, may offer a more comprehensive explanation for the heightened arousal observed in individuals with FM.

##### Sensitivity to Sensory Stimuli (Auditory, Olfactory, and Visual)

During an fMRI study, the imaging system produces loud repetitive sounds, therefore requiring participants to wear headphones or earplugs for hearing protection. However, the MRI sounds are still clearly audible. Previous studies have determined that FM patients exhibit increased reactivity to both nociceptive and non-nociceptive stimuli, suggesting heightened sensitivity to somatosensory information and possibly other sensory stimuli as well [47,48]. There have been conflicting approaches regarding whether FM patients truly experience increased sensitivity to certain sensory inputs, such as auditory stimuli, leading to elevated neural arousal [47]. Studies have explored the possibility that auditory stimuli elicit varying responses in FM. When observing the effects of varying sound intensities and tones, Samartin-Veiga et al. [49] found no evidence of increased sensitization to auditory stimuli in FM patients compared to healthy controls, even after accounting for potential confounds by varying sequences of stimuli. Overall, FM patients did not demonstrate specific patterns that indicated heightened arousal to auditory stimuli, and no significant differences in auditory sensitivity and arousal were observed amongst FM individuals compared to healthy controls [49]. Similarly, Carrillo-de-la-Pena et al. [47] predicted that FM patients would experience increased arousal due to augmented intensity of auditory stimuli based on self-reported sensitivity to nociceptive inputs. The study concluded that heightened sensory responses due to non-painful stimuli, such as auditory input, were only present during the later stages of sensory integration after prolonged exposure to these stimuli [47]. This finding suggested the possibility that increased exposure to auditory stimuli may lead to neural arousal and reactivity within FM patients. However, this increased auditory arousal was not seen to occur to a significant extent that was observed in both studies. Additionally, it remains unclear whether any observed increase in arousal was simply due to prolonged sensory integration. As a result, the significance of auditory input in fMRI studies involving FM patients remains inconclusive and is therefore not the most likely cause of the observed initial rise in BOLD responses in FM participants.

In addition to the presence of a heightened auditory response, studies have explored the possibility of other heightened sensory responses within FM, including olfactory and visual sensitivity. When focusing on olfactory and gustatory function within people with FM, it was determined that individuals have impairments in these sensations, which were additionally associated with depression [50]. When exploring the role of the visual system, studies identified heightened connectivity within visual networks [51]. It was shown that this connectivity was linked to increased fatigue, reduced emotional and cognitive processing, and additionally, symptom severity [51]. Although these studies demonstrate impairments in these sensations, it does not explain the presence of heightened arousal that is explored in the present study.

#### 3.1.2. Physiological Influences

##### BDNF and GABA Relationship

Previous studies have indicated that gray matter volume is reduced in people with FM [52]. However, the underlying causes of these reductions and the factors influencing them remain unclear. As a result, Pomares et al. [52] explored whether grey matter reductions were particularly evident across FM patients in the PC and AC and whether they were associated with gamma-aminobutyric acid (GABA) receptor concentration. It was found that decreases in the tissue water content, as indicated by T_1_ relaxation times, explained grey matter reductions, whereas increased GABA receptor concentration was found to explain grey matter increases [52]. Pomares et al. [52] questioned why grey matter decreases within certain regions were not explained by the increased presence of GABA receptors and hypothesized if neurons in areas of high GABA concentrations were likely unaffected by reductions in grey matter. Another possible explanation for the increased GABA receptor concentrations was that the upregulation of these receptors could mask the presence of neurodegeneration in FM patients [52]. However, further research is necessary to determine which of these explanations is true.

In addition to studies surrounding GABA’s relationship with grey matter volume, its role in sensitization and neural processing has also been explored. GABAergic transmission can be suppressed by the neurotrophin BDNF, leading to hyperexcitability of neural circuits in FM patients and therefore increasing their pain experience, as shown by Caumo et al. [25]. They found that BDNF plays a key role in central sensitization, explaining the significance of its reaction with GABA [25]. In FM, they found that GABA is the primary inhibitory neurotransmitter and is dysregulated, leading to heightened pain pathways and an increase in excitatory neurotransmission [25]. Therefore, this group concluded that the increased neurotransmission occurred due to elevated BDNF levels, thus weakening the inhibitory role of GABA [25]. As a result, they determined that BDNF suppresses GABA transmission, leading to an imbalance in neurotransmission of excitatory and inhibitory signaling. However, it remains unclear whether this mechanism directly contributes to FM patients’ heightened pain perception or has any relationship with the observed grey matter reductions.

##### Mitochondria Dysfunction

An additional area of interest in understanding the underlying pathology of FM is the concept of mitochondrial dysfunction, which may occur due to oxidative stress, reduced membrane potentials, disruption of mitochondrial mechanisms, or a combination of these factors. A study observing skeletal muscle alterations in FM by Inferrera et al. [53] focused on the quality control of mitochondria, revealing impairments in biogenesis, dynamics, and function. The findings indicated that FM may be characterized by changes in mitochondrial structure, a reduction in enzyme activity (specifically citrate synthase and cytochrome-c oxidase), and decreased efficiency of oxidative phosphorylation [53]. As a result, these changes can initiate increased oxidative stress markers and the production of reactive oxygen species, leading to inflammation within skeletal muscle, which may indicate a critical role in the underlying pathophysiology of FM [53]. This group also determined that mitochondrial dysfunction may occur from disruptions in proteins such as Mitofusin 2, an important protein for mitochondrial fusion, which was found to be downregulated in FM [53]. Additionally, a reduction in Coenzyme Q10 was observed, causing an impairment in the respiratory chain function and leading to oxidative stress and ATP deficiency.

Huang et al. investigated neuroinflammation and distorted neuronal plasticity in FM in relation to mitochondrial dysfunction and increased oxidative stress [28]. This study identified elevated lipid peroxidation and imbalances in antioxidant systems, including reduced glutathione levels and superoxide dismutase activity, as markers of mitochondrial dysfunction [28]. The authors found that a therapeutic agent, Boswellia Serrata, could improve mitochondrial biogenesis by promoting the nuclear translocation of the protein PGC-1α, leading to the expression of several regulatory genes [28]. These findings suggest that it is possible to treat the downstream effects of mitochondrial dysfunction with Boswellia Serrata in FM, which may be significant in identifying the underlying pathology of FM.

The presence of oxidative stress within FM is a concept explored by several researchers. Many different mechanisms have the potential to initiate oxidative stress, with mitochondrial dysfunction being a possible factor [54]. Martinez-Lara et al. analyzed blood samples and investigated mitochondrial homeostasis within FM patients compared to healthy controls [54]. This study determined that oxidative stress may have occurred due to irregular mitochondria activity, therefore demonstrating an impairment in cellular function in FM [54].

A primary structural component within mitochondria is the cristae. A study by Israel et al. [55] found that in participants with FM, the number of mitochondria in peripheral blood mononuclear cells with visibly intact cristae was reduced, and a significant number of mitochondria lacked cristae altogether. Additionally, they identified electron-dense aggregates within cells, likely ribosome aggregates, suggesting that cristae reduction and possible ribosome aggregation may be stress-induced cellular responses. These findings indicate that changes in mitochondrial morphology contribute to mitochondrial dysfunction, leading to cellular stress, such as insufficient oxidative phosphorylation and lack of ATP production. This oxidative stress may influence the widespread pain and central characteristic of FM, as well as an impaired ability to respond to cellular stress and balance metabolic demands efficiently.

##### Changes in Glial Cells

The underlying pathophysiology of FM has been explored by examining the role of glial cell activation. Recent studies suggest that autoimmune mechanisms may influence FM due to the elevated levels of cytokines present within the cerebrospinal fluid (CSF), indicating the presence of both neural and systemic inflammation (such as Fanton et al. [56]). This concept has been further investigated in relation to the pain-inducing actions of immunoglobulin G (IgG) antibodies, which bind to satellite glial cells (SGC) in the dorsal root ganglia [56]. This binding may contribute to the pathogenic mechanisms that suggest FM is an autoimmune condition. The authors of this study found that FM patients exhibited greater anti-SGC IgG levels in comparison to healthy controls, which coincided with elevated pain and a worsened disease state based on questionnaire data [56]. In the same study, magnetic resonance spectroscopy (MRS) revealed an association between elevated anti-SGC IgG levels and a reduction in specific brain metabolites in the thalamus and rostral ACC.

Another study supported the ideas of Fanton et al. [56] by examining neuroinflammation in FM patients as a result of IgG-bound SGCs. Krock et al. [57] based their study on previous research surrounding similar pain disorders, such as rheumatoid arthritis and neuropathic pain, where SGCs were found to surround sensory neuron soma and play a role in nociception and pain perception. These findings additionally demonstrated that increased anti-SGC IgG levels were linked to FM disease severity [57]. The overall findings indicate that an interaction between the peripheral nervous system and the immune system occurs in FM, as evidenced by the presence of antibodies binding to SGCs [57]. However, it remains uncertain whether anti-SGC antibodies occur due to the presence of FM or if they contribute to the disease onset. While this study established that these antibodies are associated with the severity of the disease, they were not connected to the duration of pain or FM itself [57].

##### DNA and RNA

DNA methylation plays an important role in transcription regulation and genome stability and is one of the many factors that is explored in relation to FM pathophysiology [26]. In FM, research has found DNA hypomethylation (a decrease in methylation occurrence) to occur within genes involved with stress regulation, DNA repair, the autonomic nervous system (ANS), and neuron abnormalities within the subcortex [26]. These changes in DNA methylation may result from neuroinflammatory responses within the immune system and central sensitization [26]. To determine the specific factors influencing these responses, Huang et al. [26] identified seven methylation factors that were associated with the clinical symptoms of immune dysregulation and central sensitization [26]. This study also suggested that cell death pathways are altered by FM and that methylation factors may disrupt the nervous system, causing these responses and contributing to FM symptoms [26].

MicroRNAs (miRNAs) are short, non-coding RNA molecules responsible for the regulation of gene expression, controlling a significant portion of the human genome, as well as binding to specific target gene sequences to inhibit translation [58]. Research has demonstrated abnormal miRNA expression in chronic diseases, prompting investigations into whether miRNA dysregulation plays a role in FM pathophysiology as well [58]. MiRNAs involved with immune responses and chronic inflammation have been identified in FM patients [58]. These miRNAs may regulate cytokine production and T-cell function, and their dysregulation may influence inflammatory responses that contribute to heightened nociceptive sensitivity and FM symptomology [58]. Additionally, miRNAs were identified to be involved with mitochondrial function and oxidative stress responses, in which abnormal expression could disrupt metabolic processes and further contribute to FM symptoms [58]. Other miRNAs identified in FM could be associated with central sensitization, lipid metabolism, vascular integrity, endothelial function, and the regulation of the hypothalamic–pituitary–adrenal (HPA) axis and stress response pathways [58]. Dysfunctional miRNAs may therefore influence these biological processes, highlighting their potential role in the underlying pathology and increased pain sensitivity characteristic of FM.

##### Proteins: Cytokines and Translocator Protein Prevalence

As discussed above, FM appears to be influenced by many biochemical variations that affect the underlying symptomology of this disorder. One frequently examined factor in research on FM pathophysiology is the presence of cytokines and their inflammatory influence. Backryd et al. [29] explored the role of cytokines by analyzing 90 different proteins using an advanced multiplex protein analysis to assess inflammatory markers in the CSF and plasma of FM subjects. Their findings indicate that cytokines appear to play a role in the pathology of FM. By promoting neuroinflammation and altering neural-resistant interactions, these molecules may propagate the hyperalgesia observed in FM [29]. Due to the presence of many relevant cytokines and proteins in the CSF, and its direct contact with the central nervous system (CNS), CSF is a critical substance for investigating FM pathology and the differences between FM patients and controls [29]. Chemokines, in particular, were identified, which is significant, as these proteins are expressed by neurons and glial cells for their role of synthesis in response to injury [29]. The increased expression of these chemokines may therefore signal heightened inflammation in FM subjects compared to controls.

The translocator protein (TSPO) is a mitochondrial membrane protein associated with glial cell activation and upregulation in chronic pain conditions such as FM [59]. It has therefore been explored as a possible pathological mechanism contributing to this disease [59]. Kosek et al. [59] investigated whether a functional genetic polymorphism influences the binding affinity of TSPO and subsequently increases pain symptoms in FM subjects. The study found that FM patients with the high-affinity TSPO binding genotype exhibited more severe symptoms, likely due to heightened neuroinflammation [59]. Additionally, TSPO is co-expressed with interleukin-8 in glial cells, which was identified as a marker in CSF, connecting TSPO in cerebral sensitization and neuroinflammatory signaling [59]. FMRI scans conducted on FM patients with the TSPO binding genotype demonstrated increased pain-related connectivity between the dorsolateral prefrontal cortex (dlPFC) and the frontoparietal network, areas implicated in the affective–motivational aspects of pain at heightened arousal [59]. This suggests that TSPO-mediated mechanisms amplify neural responses to pain and therefore may contribute to amplified neural responses and motivation aspects of pain within FM subjects [59].

##### Presence and Regulation of Dopamine

Multiple neurobiological factors may influence chronic pain experiences in FM. One possible factor that has been investigated is dysfunctional dopamine (DA) transmission, resulting in atypical pain perception in disorders such as FM [27]. Since DA plays a role in both nociception and cognitive function, it may contribute to the cognitive impairments frequently reported by FM patients [27]. To support this, fMRI data revealed that FM patients showed poor levels of brain activation during working memory tasks [27]. Researchers further identified dysfunctional DA function through the observation of low cortical DA receptor binding affinity and found that pain sensitivity and tolerance were associated with receptor availability [27]. Several cortical regions involved in pain processing, including the anterior cingulate cortex (ACC), orbitofrontal cortex, fusiform gyrus, and parahippocampal gyrus, exhibited reduced DA receptor binding potential in FM subjects [27]. This evidence suggests that abnormal DA regulation in FM may influence how pain is processed and perceived by patients with this disorder [27].

Further studies have explored the role of dopaminergic neurons in potential pain-related functions, including perception, severity, anticipation, emotional responses, and regulation [60]. Katar et al. [60] therefore examined the relationship between DA and niacin, a B vitamin potentially involved in mechanisms such as central sensitization, immune function, neuroendocrine responses, and additional nutritional factors. Since a coenzyme of niacin is involved in the limitation of DA synthesis, this relationship is significant [60]. Their study found that serum niacin and DA levels were reduced in FM subjects, leading to insufficient DA synthesis and increased nociceptive sensitivity and pain perception [60]. Additionally, research has found that not only DA metabolites but also serotonin and norepinephrine are reduced in the CSF of FM patients compared to controls [60]. This finding therefore suggests a metabolic dysfunction that affects neurotransmitter and hormone receptor regulation, ultimately contributing to increased neural arousal and pain perception [60].

Another important role of DA is its role in the promotion of neuron integrity [61]. A common finding in FM research is the reduction in grey matter density in FM subjects compared to controls. Wood et al. [61] explored a possible mechanism underlying grey matter reductions by analyzing DA metabolism within the limbic cortex and mesencephalon. This study investigated whether a relationship exists between DA metabolism and grey matter density, ultimately providing evidence that DA plays a protective role within the limbic cortex [61]. As a result, a correlation was found between corticolimbic DA metabolism and grey matter density in the parahippocampal gyri and pregenual cortex was observed amongst FM subjects [61]. Both of these cortical areas receive DA signals and regulate their activity, indicating that abnormalities in DA metabolism may contribute to FM pathology [61].

### 3.2. Study Component 2: Analysis of FMRI Data

**Figure 4:**
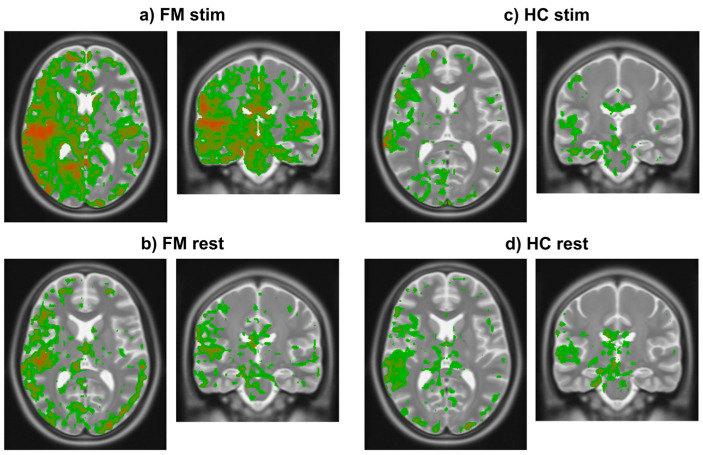
Images in radiological orientation depicting the magnitude of the initial rise in BOLD signal in each voxel averaged within each of the four groups. (**a**) Transverse (**left**) and coronal (**right**) slices showing results in the FM stim (n = 84) group/condition. The slice locations were centered on the highest signal change region in this group. (**b**) Transverse and coronal slices at the same locations as in frame a in the FM rest (n = 79) group. (**c**) Corresponding transverse and coronal slices showing results in the HC stim (n = 63) group. (**d**) Again, corresponding slices in the HC rest (n = 66) group. Colors indicate the magnitude of the initial rise response with red being highest, decreasing through orange, then yellow, and green being the lowest values above the chosen threshold.

The results of analyzing existing fMRI data demonstrated that the initial rise in BOLD occurs consistently across study groups (HC, FM) and conditions (stim, rest) (Figure 4). Using a threshold of 0.25% signal change, the results demonstrate that the initial rise occurs only in grey matter regions. The initial rise is also larger in specific regions of the brain. Comparisons of the FM stim group to the FM rest, HC stim, and HC rest groups demonstrate significant differences (unpaired two-tail *t*-test) between groups. Regions of interest (ROIs) were selected as those that displayed an average BOLD signal rise above 0.25% and ignoring any confounds of extreme changes over 3% that may have occurred as an artifact within the fMRI data. The mean rise and standard deviation (std) for each region were then calculated, as seen in Table 1. The resulting images demonstrate where the heightened BOLD responses occurred, using a color scale spanning from blue at the most negative values to green near zero and increasing to red at the highest values (Figure 4).

The mean and standard deviations of the initial rise values in the ROIs for each study group/condition (Table 1) demonstrate that the values are larger in the FM stim group than in the other conditions. The regions with the largest initial rise in BOLD signal in the FM stim condition (Figure 4a) are the right insular cortex (IC) (0.35 ± 0.30, expressed as mean ± std), posterior cingulate cortex (PCC) (0.34 ± 0.26), anterior cingulate cortex (ACC) (0.34 ± 0.32), periaqueductal grey (PAG) (0.48 ± 0.49), parabrachial nucleus (PBN) (0.40 ± 0.35), thalamus (0.33 ± 0.32), frontal orbital cortex (Forb) (0.27 ± 0.25), hypothalamus (0.29 ± 0.36), and amygdala (0.30 ± 0.37). This evidence suggests that the regions with the most significant BOLD signal increases may play a role in heightened pain sensitivity in FM.

The results of the *t*-test comparisons between groups provide evidence that the FM rest condition has a significant difference in BOLD signal rise compared to FM stim (Table 2). The comparisons show significant differences in the IC (*p* = 1.31 × 10^−3^, 0.22 ± 0.26), PCC (*p* = 4.80 × 10^−5^, 0.17 ± 0.30), ACC (*p* = 1.39 × 10^−3^, 0.19 ± 0.31), PAG (*p* = 4.43 × 10^−4^, 0.24 ± 0.38), thalamus (*p* = 4.42 × 10^−3^, 0.20 ± 0.27), and PBN (*p* = 5.82 × 10^−4^, 0.21 ± 0.39). Regions that did not reach statistical significance (*p* < 0.05) included the FOrb (0.19 ± 0.27), hypothalamus (0.15 ± 0.45), and amygdala (0.18 ± 0.48). Furthermore, the difference in signal rise seen within these two conditions in the FM group suggests that participants may have anticipated the condition of each trial sufficiently accurately to influence the group’s average results.

When comparing the HC stim condition to the FM stim, the IC (*p* = 1.95 × 10^−4^, 0.18 ± 0.24), PCC (*p* = 3.67 × 10^−6^, 0.14 ± 0.26), ACC (*p* = 5.79 × 10^−5^, 0.15 ± 0.26), and thalamus (*p* = 5.35 × 10^−4^, 0.16 ± 0.32) demonstrated statistically significant differences (Table 2). Significant differences were not indicated within the FOrb (0.16 ± 0.28), PAG (0.29 ± 0.47), hypothalamus (0.18 ± 0.33), PBN (0.29 ± 0.32), and amygdala (0.14 ± 0.32).

In comparison to the FM stim condition, the HC rest condition exhibited significantly different values in the IC (*p* = 4.65 × 10^−4^, 0.20 ± 0.25), PCC (*p* = 1.80 × 10^−4^, 0.19 ± 0.26), FOrb (*p* = 6.57 × 10^−4^, 0.14 ± 0.25), and ACC (*p* = 1.32 × 10^−4^, 0.16 ± 0.84) (Table 2). Regions without evidence of statistically significant differences included the PAG (0.33 ± 0.43), hypothalamus (0.22 ± 0.33), thalamus (0.23 ± 0.29), PBN (0.29 ± 0.33), and amygdala (0.22 ± 0.34).

When comparing the signal rise magnitudes in the HC rest and stim conditions as compared to FM rest (Table 3), there were no significant differences in the regions of interest. These same results were determined when observing the difference in this signal between HC rest and HC stim (Table 4).

## 4. Discussion

Possible explanations for the observation of BOLD signal rises in FM participants and thus increased metabolic demand were investigated by means of a literature review and re-analysis of existing fMRI data sets. The findings indicate that several possible mechanisms may contribute to the increased metabolic demand, including both physiological and psychological factors. The initial rise in BOLD signal is shown to occur specifically in grey matter regions and is more pronounced in certain areas of the brain. The signal increases were also more pronounced in the FM stim group/condition in advance of noxious heat stimuli being applied.

### 4.1. Interpretation of Literature Review

The results of the literature review indicate that it is probable that no single mechanism is solely responsible for the observed physiological and psychological factors influencing the increased arousal in FM patients. This is particularly evident, as many potential mechanisms have been determined to contribute to this condition. For example, miRNA has been found to influence pathological mechanisms that may contribute to FM symptomology [58]. Possible dysfunctions associated with FM include the dysregulation of the immune system, mitochondrial dysfunction, reduced vascular function, central sensitization, or HPA dysfunction [58]. Additionally, neuroinflammatory mechanisms, particularly when involving the increased presence of cytokines, have been studied in relation to FM and chronic pain [29]. Elevated levels of inflammation-related proteins in CSF and plasma further suggest that FM should be studied further as a physiological condition rather than being attributed solely to psychological factors [29].

Additionally, FM has been proposed to arise from mitochondrial dysfunction and oxidative stress, thus raising the question of whether there are specific proteins correlated with mitochondrial homeostasis in muscle tissue and therefore linked to FM etiology [53]. This stems from the concept that FM is characterized by reduced movement, muscular sensitivity, and long-lasting pain. Additionally, people with FM have muscle tissue abnormalities potentially arising from pathological issues such as increased DNA fragmentation, mitochondrial alterations, and issues regarding essential enzymes [53]. Similarly to other neurodegenerative disorders such as Parkinson’s disease, DA has been found to play a significant role in neuron integrity [61]. Previous imaging studies have also revealed reductions in grey matter volume in FM patients, which may be contributing to their psychological and physiological symptoms [61]. As a result, researchers are actively investigating a pathological basis for these findings, whether they stem from alterations in DA metabolism or GABA affecting neuronal integrity [52]. Another explanation that has been investigated regarding the signal rise observed in FM participants involves changes in serum neurotrophins such as BDNF, which is predicted to influence motor cortex disinhibition, as well as the function of descending pain modulation [25].

One can interpret from these results that there are numerous possibilities that may contribute to FM symptomology and to the initial rise in BOLD signal that has been observed to occur in specific grey matter regions of the brain. Additionally, it is unclear which of these possible influences are causes and which are effects.

Oxidative stress appears to be a key factor underlying various pathological influences, including mitochondrial dysfunction, neuroinflammation caused by increased cytokine presence, miRNA dysregulation, and elevated cortisol levels resulting from psychological distress. Mitochondria dysfunction can trigger an increase in oxidative stress markers and reactive oxygen species, consequently influencing cellular function and neuroinflammation [53]. Additionally, studies have shown a connection between mitochondria dysfunction and DA-related neurodegeneration, as depicted by grey matter reductions in FM patients [62]. If cytokine levels are elevated and miRNA regulation is impaired, inflammatory responses may be exacerbated, further contributing to mitochondrial dysfunction and oxidative stress [58]. As a result, the oxidative stress may have contributed to irregular neural signaling as observed in the form of the initial rise in BOLD signal. If oxidative stress occurs due to heightened mitochondrial dysfunction, it has the potential to disrupt neural signaling at the cellular level, again possibly explaining the initial rise observed in the fMRI data. Furthermore, stress induced by study trials, combined with oxidative stress-related irregular signaling, could have played a role in the observed BOLD signal increases.

Another possible explanation for the initial rise suggested by the results of the literature review is that FM participants may exhibit dysfunction within their ANS. The ANS is responsible for its widespread innervation throughout the body, particularly for maintaining a state of homeostasis in response to any internal or external stressors and consequently regulating both physiological and psychological mechanisms [63]. It is possible that these mechanisms are interconnected, together contributing to the symptomology of FM as well as the metabolic demand as exhibited by the initial rise. Additionally, symptoms may be further amplified by psychological distress rather than solely the possible physiological factors as controlled by the ANS. Chronic pain imposes a significant psychological burden and induces considerable stress in individuals with FM, leading to elevated levels of stress hormones such as cortisol [44]. It is possible that individuals with FM experience ANS dysfunction, therefore leading to a state of constant arousal—in “fight or flight” mode—leading to neuroinflammation and oxidative stress as a coping mechanism to maintain homeostasis. However, instead of regulating the nervous system, it may have led to heightened nociception and stress. Therefore, this could be further amplified due to any stress that occurs during the trials throughout the study, thus causing the initial rise. Overall, this literature review suggests that many of these physiological and psychological mechanisms that have been found by previous research are interconnected, may be influencing one another, and may occur due to dysfunction within the ANS, ultimately leading to heightened pain perception in FM patients.

### 4.2. Interpretation of fMRI Data Analysis

Findings from the fMRI data analysis revealed that the signal rise was more pronounced in the FM stim group compared to the FM rest, HC stim, and HC rest conditions. As hypothesized, this signal was localized within grey matter regions. This is significant, as grey matter contains cell bodies, thus indicating that where this initial rise is occurring is where neural metabolic activity occurs and is therefore not attributable to image artifacts, participant movement, data processing errors, or other confounds. The regions depicting the most notable increases in BOLD response prior to stimulation onset were observed in the IC, ACC, PCC, PAG, PBN, and thalamus. Although no significant difference was found in the signal amongst the FM rest and HC conditions, these regions identified are likely involved in initiating the heightened arousal experienced by FM participants. This response may be influenced by increased stress during trials and anticipation associated with the stimulation condition, potentially explaining the intensity of the signal amongst the FM stim group. Consequently, these results indicate that if the anticipation of the stimulation condition affects the initial rise, then it must be affected by the person’s cognitive state.

The right posterior IC exhibited the largest increase in BOLD signal indicating a heightened state of metabolic demand in this region, particularly in the FM stim group. The insula integrates sensory information from various regions and can be divided into two functional areas: the anterior insula, which regulates the emotional and motivational components of pain, and the posterior insula, which processes sensory and contextual stimuli [64]. The posterior IC is particularly involved with interoceptive signals, including pain, temperature, muscular and visceral feelings, and vascular dilation [65]. Notably, the right insula plays a critical role in regulating negative emotions such as pain [64]. Therefore, the increased activation of the right posterior IC may reflect a heightened response to chronic pain in FM participants in association with possible negative and stress-filled associations these participants may have with the fMRI trials within this study. If the participants experienced states of increased stress, discomfort, or even pain catastrophizing, this state of arousal within the IC could be explained. Bergeron et al. [66] determined that the posterior insula is one of the few brain regions capable of eliciting pain when stimulated by low-frequency electrical stimulation, further revealing how information and signals in the spinothalamic pathway are processed by observing connections between the thalamus and the posterior insula [66]. This study therefore highlighted the relationship between structures involved in pain processing and the heightened response observed in FM stim participants [66]. As a result, it can be interpreted that the posterior IC is involved in pain processing as well as negative emotions such as stress, therefore being a potential cause of the initial rise in the BOLD signal.

The ACC is involved with various cognitive and emotional processes, including attention, memory consolidation, and anxiety [67]. Previous research has found that systemic inflammation can alter neural activity within regions such as the ACC, thus affecting neurotransmitter sensitivity and subsequent behavior [67]. These findings suggest that inflammation may drive changes in neural activity and behavior, potentially causing adaptations of signaling between regions such as those observed in the current study [67]. Therefore, it can be interpreted that the ACC involvement with stress and anxiety may be apparent via the heightened signaling in the FM stim group. Additionally, the neuroinflammation that is apparent in FM may be further contributing to increases in this signal.

Findings from the data analysis further demonstrated significant signaling within the PCC and thalamus—two structures that share a strong integrative relationship. Research has highlighted their critical role in processing stress, particularly in the context of pain anticipation [68]. This finding is particularly relevant in the context of the current study, in which it could explain the initial rise. Warren et al. [22] found that connectivity between the PCC and thalamus was increased in FM participants, suggesting that heightened pain stimulation and arousal are unique to FM, as seen through the increased activation of these regions. Although, the precise function of the PCC remains uncertain, prior research suggests that it may play a central role in the default mode network, contributing to attention, saliency, memory, and even pain processing and catastrophizing [22]. If this is the case, the heightened activation within the FM group may result from increased anticipation of painful stimuli, the physical memory of prior stimuli, or even heightened awareness of the noise and general discomfort with the fMRI environment.

The PBN is another structure found to be central to processing aversive stimuli and pain perception [69]. In this study, it was found to be significantly active in both FM groups, suggesting increased nociceptive responses even in the absence of painful stimuli—a pattern not observed in either HC group. Threat memory and fear conditioning are additional aversive behaviors that are associated with the PBN, in addition to its role in nociception [69]. Previous research has linked chronic pain to deficits in affective processing within the PBN, leading to increased symptoms of depression and anxiety [69]. Further findings have demonstrated that PBN neurons responding to noxious heat can become conditioned to respond to auditory stimuli, such as an MRI system’s sounds [69]. Therefore, it is possible that FM participants formed aversive associations between the auditory stimuli of the fMRI environment and the heat stimuli presented in the stimulation trials. Given that FM patients often exhibit heightened salience and memory of pain, PBN neurons may have been hyperactivated due to a conditioned pain response. Or, alternatively, the PBN may be active simply due to the increased nociceptive stimuli experienced by FM participants and was not further activated by the conditions of the study.

Another critical structure involved in nociceptive signal processing and the descending pain system is the PAG [70]. Research indicates that emotional pain, such as depression, shares similar symptomology and pathophysiology with chronic pain [71]. The PAG plays a significant role in pain regulation and has been implicated in the comorbidity of pain and depression, as these conditions can exacerbate each other’s effects [71]. Additionally, this structure plays an essential role in the maintenance of the brain’s stress response [71]. Stress has been found to activate endogenous pain systems, thus influencing pain perception based on the stress state of an individual [71]. Studies have provided evidence of altered functional connectivity within the PAG of individuals with chronic pain [72]. The PAG has additionally been found to be connected and receive direct information from areas such as the ACC and IC [72]. Given these regions are implicated in pain perception, alterations in their neural pathways may contribute to the heightened nociceptive sensitivity observed in chronic pain conditions [72]. These findings align with the current data analysis, suggesting that heightened stress or an aversive state within structures like the ACC and IC may alter neural pathways, resulting in increased arousal in the FM stim group prior to stimulus activation.

Based on the findings of this study, it can be speculated that participants may have been able to predict the stimulation conditions during each fMRI trial. Given the neurological regions found to be activated, the heightened stress associated with the stimulation trials may have contributed to a sympathetic response, placing participants in a state of increased stress. Consequently, FM participants, who are experiencing chronic pain, may have had their symptoms amplified due to this heightened state of stress and arousal in conjunction with their sensitivity to nociceptive stimuli.

### 4.3. Limitations and Future Directions

Like any study, there are certain limitations of this study to be acknowledged and taken into account for future research. Firstly, despite the extensive research involved in the literature search, it is possible that some mechanisms were overlooked and may be relevant. It is difficult to determine every possible factor that could exist; therefore, some unexamined physiological aspects may also contribute to the etiology of FM or the observed initial rise. Another limitation is the innate complexity of neural activity, as it is not expected that entire anatomical regions demonstrate uniform BOLD responses, as there are sub-regions. Nonetheless, the comparison observed in the data analysis shows differences in responses across groups for the exact same groups of voxels in the data and shows how responses vary across whole regions. Additionally, if participants were truly capable of predicting the study type of each trial and therefore anticipating the stimulus, this would also be considered a limitation. Therefore, future studies should explore methods to eliminate this to further interpret the meaning of these results.

Based on the findings of this study, future research should explore possible connections between the heightened arousal evident in FM stim participants (within the IC, ACC, PCC, PAG, PBN, and thalamus) and the physiological mechanisms identified in the literature review. Given that this increased activation appears to result from a state of increased stress, future studies should particularly explore the role that ANS dysfunction, oxidative stress, and mitochondrial dysfunction play in initiating this response within FM. Another question that could be answered through additional research is whether this initial rise occurs as a result of pain or if it is the cause of the heightened pain in FM. By answering these questions, we may be able to better understand this chronic pain disorder and how to treat it.

## 5. Conclusions

This study highlights the complex relationship that exists between physiological and psychological mechanisms within FM, particularly the heightened neural arousal observed in response to anticipated stimuli. The increased activation of key brain regions, including the IC, ACC, PCC, PAG, PBN, and thalamus, suggests that pain perception in FM is not solely a response to nociceptive input but is also influenced by stress, anticipation, and neural dysregulation. The studies to date provide evidence of physiological dysregulation within mechanisms such as gene expression, oxidative stress, mitochondrial dysfunction, and cytokine activity that may be underlying mechanisms of FM. It is not clear how, or if, these processes are related and which are causes and which are effects. However, it appears that these factors could explain altered levels of neurotransmitters and proteins, such as DA, GABA, and BDNF, which in turn may explain altered neuronal integrity resulting in the observed reduction in gray matter volume. In addition, it has been shown that BDNF plays a key role in central sensitization and suppresses GABA transmission, which could explain heightened neural excitability and altered connectivity between brain regions in FM. Combined, the studies to date therefore suggest that FM involves metabolic dysfunction and neuroinflammation that affects neurotransmitter, protein, and hormone regulation, ultimately contributing to increased neuronal excitability, possible dysregulation of stress responses, and heightened pain perception. The initial rise in BOLD responses observed during fMRI runs may reflect the dysregulated stress responses.

Understanding these interconnected mechanisms provides valuable insight into the etiology of FM and may contribute to the development of targeted interventions aimed at lessening symptoms as a result of increased stress. Future research should explore the underlying physiological factors of these neural dysfunctions and investigate methods to reduce stress sensitivity in FM patients, potentially leading to more effective treatments and therefore reducing pain. By addressing both the neural and systemic factors underlying FM, this research contributes to a more rounded understanding of chronic pain disorders. Moreover, the current findings may lead to the identification of biomarkers that can be used to diagnose FM.

## Figures and Tables

**Table 1:** Average size (mean ± standard deviation) of the BOLD signal increases at the onset of fMRI runs for each brain region.

Brain Region	FM Stim	FM Rest	HC Stim	HC Rest
IC	0.35 ± 0.30	0.22 ± 0.26	0.18 ± 0.24	0.20 ± 0.25
PCC	0.34 ± 0.26	0.17 ± 0.30	0.14 ± 0.26	0.19 ± 0.26
FOrb	0.27 ± 0.25	0.19 ± 0.27	0.16 ± 0.28	0.14 ± 0.25
ACC	0.34 ± 0.32	0.19 ± 0.31	0.15 ± 0.26	0.16 ± 0.28
PAG	0.48 ± 0.49	0.24 ± 0.38	0.29 ± 0.47	0.33 ± 0.43
Hypothalamus	0.29 ± 0.36	0.15 ± 0.45	0.18 ± 0.33	0.22 ± 0.33
Thalamus	0.33 ± 0.32	0.20 ± 0.27	0.16 ± 0.26	0.23 ± 0.29
PBN	0.40 ± 0.35	0.21 ± 0.39	0.29 ± 0.32	0.29 ± 0.33
Amygdala	0.30 ± 0.37	0.18 ± 0.48	0.14 ± 0.32	0.22 ± 0.34

**Table 2:** Comparisons of BOLD initial rise values between the FM stim group/condition and FM rest, HC stim, and HC rest (two-tailed Student’s *t*-tests). Bolded values indicate regions of statistical significance, *p* < 0.0056 (*p* < 0.05 corrected for 9 comparisons) df: degrees of freedom.

Brain Region	FM Rest (df = 161)		HC Stim (df = 145)		HC Rest (df = 148)	
	*t*	*p*	*t*	*p*	*t*	*p*
IC	**3.06**	**1.31 × 10^−3^**	**3.63**	**1.95 × 10^−4^**	**3.38**	**4.65 × 10^−4^**
PC	**4.00**	**4.80 × 10^−5^**	**4.65**	**3.67 × 10^−6^**	**3.65**	**1.80 × 10^−4^**
FOrb	1.96	2.61 × 10^−2^	2.48	7.19 × 10^−3^	**3.28**	**6.57 × 10^−4^**
AC	**3.04**	**1.39 × 10^−3^**	**3.96**	**5.79 × 10^−5^**	**3.74**	**1.32 × 10^−4^**
PAG	**3.39**	**4.43 × 10^−4^**	2.26	1.25 × 10^−2^	1.93	2.75 × 10^−2^
Hypothalamus	2.26	1.25 × 10^−2^	1.92	2.87 × 10^−2^	1.23	1.10 × 10^−1^
Thalamus	**2.65**	**4.42 × 10^−3^**	**3.34**	**5.35 × 10^−4^**	1.9	2.97 × 10^−2^
PBN	**3.31**	**5.82 × 10^−4^**	2.04	2.18 × 10^−2^	2.06	2.03 × 10^−2^
Amygdala	1.69	4.69 × 10^−2^	2.56	5.78 × 10^−3^	1.25	1.06 × 10^−1^

**Table 3:** Comparisons of BOLD initial rise values between the FM rest group/condition and HC stim and HC rest (two-tailed Student’s *t*-tests).

Brain Region	HC Stim (df = 140)		HC Rest (df = 143)	
	*t*	*p*	*t*	*p*
IC	0.73	0.23	0.45	0.33
PC	0.57	0.28	−0.41	0.66
FOrb	0.61	0.27	1.25	0.11
AC	0.9	0.19	0.7	0.24
PAG	−0.74	0.77	−1.29	0.90
Hypothalamus	−0.49	0.69	−1.11	0.87
Thalamus	0.94	0.17	−0.58	0.72
PBN	−1.27	0.90	−1.25	0.89
Amygdala	0.53	0.30	−0.55	0.71

**Table 4:** Comparisons of BOLD initial rise values between the HC stim group/condition and HC rest (two-tailed Student’s *t*-tests).

Brain Region	HC Rest (df = 127)	
	*t*	*p*
IC	−0.28	0.61
PC	−1.01	0.84
FOrb	0.56	0.29
AC	−0.19	0.58
PAG	−0.43	0.66
Hypothalamus	−0.71	0.76
Thalamus	−1.42	0.92
PBN	0.03	0.49
Amygdala	−1.30	0.90

## Data Availability

The data used in this study were from previous studies and are available upon reasonable request from the corresponding author. The analysis software, Pantheon, is freely available on GitHub at https://github.com/stromanp/pantheon-fMRI (accessed on 29 May 2025).
